# The role of thrombin in the pathogenesis of diabetic neuropathy

**DOI:** 10.1371/journal.pone.0219453

**Published:** 2019-07-05

**Authors:** Efrat Shavit-Stein, Ramona Aronovich, Constantin Sylantiev, Shany Guly Gofrit, Joab Chapman, Amir Dori

**Affiliations:** 1 Department of Neurology, Sheba Medical Center, Tel HaShomer, Israel; 2 Joseph Sagol Neuroscience Center, Sheba Medical Center, Tel HaShomer, Israel; 3 Department of Physiology and Pharmacology, Sackler Faculty of Medicine, Tel Aviv University, Tel Aviv, Israel; 4 Department of Neurology, Sackler School of Medicine, Tel Aviv University, Tel Aviv, Israel; 5 Robert and Martha Harden Chair in Mental and Neurological Diseases, Sackler Faculty of Medicine, Tel Aviv University, Tel Aviv, Israel; 6 Talpiot medical leadership program, Sheba Medical Center, Tel HaShomer, Israel; Medical University Innsbruck, AUSTRIA

## Abstract

Diabetic neuropathy is common and disabling despite glycemic control. Novel neuroprotective approaches are needed. Thrombin and hypercoagulability are associated with diabetes and nerve conduction dysfunction. Our aim was to study the role of thrombin in diabetic neuropathy. We measured thrombin activity by a biochemical assay in streptozotocin (STZ)-induced diabetic neuropathy in male Sprague-Dawley rats. Neuropathy severity was assessed by thermal latency and nerve conduction measures. Thermal latencies were longer in diabetic rats, and improved with the non-specific serine-protease inhibitor Tosyl-L-lysine-chloromethyl ketone (TLCK) treatment (p<0.01). The tail nerve of diabetic rats showed slow conduction velocity (p˂0.01), and interestingly, increased thrombin activity was noted in the sciatic nerve (p˂0.001). Sciatic nodes of Ranvier and the thrombin receptor, protease activated receptor 1 (PAR1) reactivity showed abnormal morphology in diabetic animals by immunofluorescence staining (p<0.0001). Treatment of diabetic animals with either the specific thrombin inhibitor, N-alpha 2 naphtalenesulfonylglycyl alpha-4 amidino-phenylalaninepiperidide (NAPAP) or TLCK preserved normal conduction velocity, (p˂0.01 and p = 0.01 respectively), and prevented disruption of morphology (p˂0.05 and p˂0.03). The results establish for the first time an association between diabetic neuropathy and excessive activation of the thrombin pathway. Treatment of diabetic animals with thrombin inhibitors ameliorates both biochemical, structural and electrophysiological deficits. The thrombin pathway inhibition may be a novel neuroprotective therapeutic target in the diabetic neuropathy pathology.

## Introduction

Diabetes mellitus (DM) is a common health problem in the western world [1] and diabetic peripheral neuropathy (DPN) affects 60% of all DM patients [2]. DPN is characterized by abnormal sensory and motor symptoms with abnormal nerve conduction measures. Nerve damage is manifested by a progressive distal-to-proximal degeneration of peripheral nerves which is related to prolonged exposure to high blood glucose [3]. The high prevalence of this complication and the absence of pharmacological therapeutics enhance the need for understanding of the underling involved mechanisms in order to develop novel and targeted drugs.

Thrombin is key coagulation factor and is known to participate in other cellular processes including inflammation [4], and neuronal degeneration [5]. Thrombin pathway is activated by a number of G-protein coupled protease activated receptors (PARs), including PAR1, which is present on glia and neurons [6]. In the peripheral nervous system (PNS) thrombin was previously shown to act in neurogenic inflammation, pain, motor function and nerve injuries [7]. We have previously demonstrated that thrombin pathway activation in the peripheral nerve induces a conduction block at the node of Ranvier. This implies a possible thrombin pathway involvement in peripheral nerve disease such as diabetic neuropathy. Indeed, thrombin is increased in the blood of diabetic patients. High thrombin levels have been linked to poor diabetic control [8]. Platelet aggregation was reported to be higher in patients with diabetic neuropathy. These patients showed capillary pathology as well. Involvement of coagulation abnormalities in the pathogenesis of diabetic neuropathy was suggested before [9].

We here examined the involvement of the thrombin pathway in the model of streptozotocin (STZ) induced hyperglycemia. This is a well-established *in vivo* diabetes model with early functional and biochemical abnormalities that are similar to those seen in human diabetic neuropathy [10]. Acute onset of diabetes is accompanied by an abrupt 20% decrease in nerve conduction velocity (NCV) followed by a more gradual but progressive further decline [11]. We examined thrombin pathway using two thrombin inhibitors; the none-specific serine protease inhibitor Tosyl-L-lysine-chloromethyl ketone(TLCK), and the selective thrombin inhibitor N-alpha 2 naphtalenesulfonylglycyl alpha-4 amidino-phenylalaninepiperidide (NAPAP). TLCK irreversibly inhibits a wide variety of serine proteases and was used in highest doses possible without causing a systemic toxicity. NAPAP on the other hand, has a specific inhibitory effect on thrombin in the dose that was used in our study [12]. The use of both inhibitors allowed to examine the specific role of thrombin activity apart from other serine proteases which may also rise during inflammatory conditions.

In the present study we found that thrombin activity is increased in the peripheral nerves of diabetic rats. We further established the pathogenic importance of this pathway by demonstrating a beneficial effect of thrombin inhibition in the experimental diabetic neuropathy model.

## Methods

### Animals

Eight weeks old male Sprague-Dawley rats (RRID:RGD_734476), weighing 250-270g (Harlan, Jerusalem, Israel) were kept under standard conditions of 23±1°C with a 12-h light-dark cycle and access to food and water ad libitum, and were acclimatized for one week prior the experiment. After diabetes induction, animals were kept in separated cages due to elevated urination rate. Animals’ health measures were assessed on daily basis (weight, mobility, ability to move freely around the cage and the condition of the fur). The animals expected weight loss was approved, since the STZ diabetic animal model is well known and accepted.

### Ethical considerations

All experiments were approved by the Tel-Aviv University Animal Welfare Committee (approval number M-06-004) and appropriate measures to avert pain and suffering to the rats were taken. Experiments were carried out according to the ARRIVE guidelines for animal research.

### Induction of diabetes

Streptozotocin (STZ, Sigma) 65 mg/kg dissolved in fresh 0.1N citrate buffer, pH 4.5 was delivered by intraperitoneal (IP) injection as previously described [13,14]. Age-matched non-diabetic rats were injected with citrate buffer alone. Diabetes was confirmed by 2 repeated measures of blood glucose levels conducted during the week following STZ injection (above 250 mg/dl) with a commercial glucometer device (Accutrend; Boehringer Manheim GmbH). Body weight of all animals was measured weekly. The animals were randomly injected with STZ. After induction, the animals were randomly allocated to different groups.

### Treatment protocol for TLCK and NAPAP

Six weeks after induction of diabetes the animals were divided into three groups that consisted a similar initial average body weight and measured blood glucose level.

Rats were treated daily for two weeks with intraperitoneal (IP) injections of N-Tosyl-Lysine-chloromethyl-ketone (TLCK-616382, Calbiochem, La Jolla, California) 4.4 mg/kg or N-alpha 2 naphtalenesulfonylglycyl alpha-4 amidino-phenylalaninepiperidide (NAPAP 76308, Fluka) 7.7μg/kg. The dose of NAPAP treatment was determined based on the range at which a specific inhibition of thrombin is achieved [12]. TLCK dose was chosen based on the highest possible dose in which there was no systemic bleeding toxicity. This was evaluated at preliminary studies. Control rats were treated with saline alone.

### Thrombin activity measure in the sciatic nerve

Thrombin levels were measured using an *ex-*vivo method. A fluorometric assay which measures cleavage of the synthetic peptide substrate N-p-Tos-Gly-L-Pro-L-Arg-7- amido-4-methylcoumarin (tos-GPR-AMC; Sigma T 0273) was employed [15]. One centimeter of sciatic nerve stripped from epineurium was immersed in 140 μl of phosphate buffer saline (PBS) with 0.1% BSA and 10 μM substrate. Substrate cleavage was measured continuously during twenty minutes at 37°C (excitation 360/40 nm, emission 460/40 nm) and compared to calibrated purified human thrombin concentrations (Sigma T 0553; 300 units/mg protein). TLCK or NAPAP (1mM and 1μM, respectively) were separately added to selected test wells fifteen minutes prior to the substrate. (Control-saline n = 3, control-NAPAP n = 3, control-TLCK n = 3, diabetic-saline n = 3, diabetic-NAPAP n = 3, diabetic-TLCK n = 3).

### Hot plate test

At seven weeks following STZ injection (1 week after starting the treatments) animals were placed in a perspex cylinder on heated stage maintained at 50±1°C. Time to heat response indicated by hind paw licking, shaking or jumping was measured. A maximum on-plate time was set to 30 seconds to prevent skin injury. (Control n = 8, diabetic saline treated n = 8, TLCK treated n = 7, NAPAP treated n = 8).

### Electrophysiological studies

Electrophysiological tests were performed eight weeks after STZ induction by a blinded tester (the measurements were performed after two weeks of treatments were completed) and as previously described [16]. Electrophysiological studies were conducted on the rats tails, in order to minimize the effect of local inflammation which is present in sciatic nerve of STZ rats [17,18]. Tail evaluation was chosen for this study as measurements are more accurate. Tails were kept in a constant temperature using a heating lamp and mat. Animals were anesthetized with Phenobarbital (IP, 24mg/kg). Compound muscle action potential (CMAP) responses were recorded using a pair of ring electrodes, with the active electrode positioned 5 cm distal to the tail base (Key point, Dantec, Skovlunde, Denmark). Two pairs of monopolar needle electrodes were used to stimulate the tail nerves. The stimulating cathode was applied 4 cm distal to the tail base and at the tail base for distal and proximal stimulation, respectively. A ground electrode was placed between the stimulating and recording electrodes. Distal and proximal latencies were measured and motor nerve conduction velocity (MNCV) was calculated per measured distance between stimulating cathodes. CMAP amplitudes were measured from the baseline to the negative peak (amplitude and NCV, control n = 4, diabetic n = 5, TLCK treated n = 5/8, NAPAP treated n = 5. Latency control n = 4, diabetic n = 5, TLCK treated n = 7, NAPAP treated n = 5).

### Localization of PAR1 in sciatic nerve slices

Sciatic nerves were fixed overnight in 4% PFA in 0.1M phosphate buffer, pH 7.4, and cryoprotected in 30% sucrose and snap-frozen. Longitudinal sections (50μm) were mounted on slides and blocked for one hour in PBS containing 1% goat serum, 0.2% glycine and 0.1% Triton X-100. Sections were then incubated overnight with rabbit anti PAR1 antibody (1:50) and mouse monoclonal anti Caspr, (1:500; a generous gift of Professor Elior Peles) in blocking solution. After rinses with PBS, sections were incubated with RRX- and Cy2-coupled secondary antibodies (Jackson Immuno Research Laboratories) in blocking solution. Immunofluorescence slides were viewed and analyzed using a confocal ZEISS CLSM 410 microscope.

### Statistical analysis

All analyses and assessments of the normality of data were carried out using GraphPad software (GraphPad Prism for Windows, GraphPad Software, La Jolla California USA). Clinical scores were assessed by analysis of variance (ANOVA) with repeated measures prospectively comparing each treatment group with STZ or controls. One-way ANOVA with Dunnett’s post hoc test was used to compare the means of TLCK, NAPAP and saline-treated controls in the histology, biochemical, and electrophysiological measures. All statistical analyses were performed on the relevant groups.

## Results

### Thrombin activity ex-vivo

**Fig 1:**
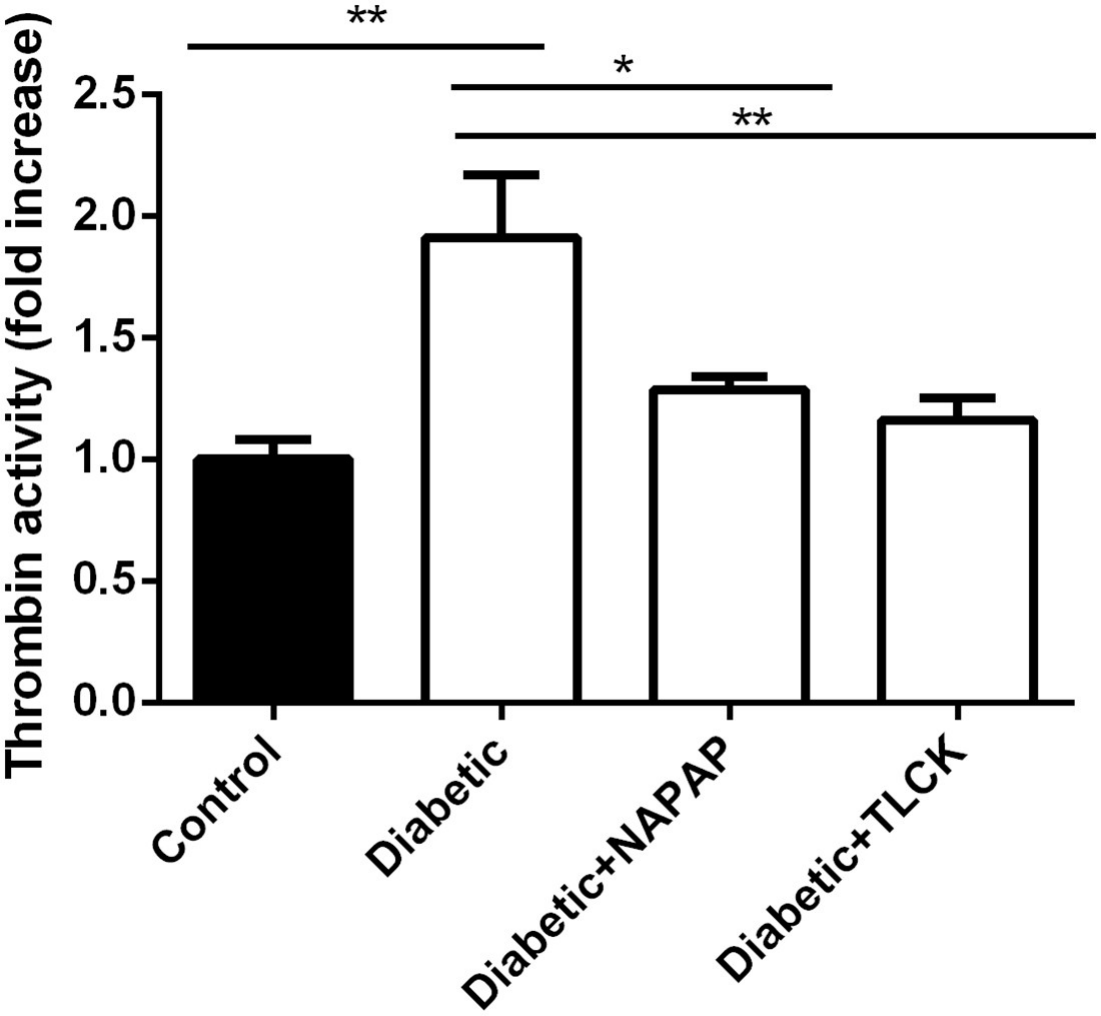
Thrombin activity is increased in diabetic sciatic nerve. An ex-vivo experiment in which thrombin inhibitors were added to the test wells. Control column includes healthy rats treated with saline, TLCK and NAPAP. Data are represented in comparison to measured thrombin activity in control column, which was defined as 1. Thrombin activity was significantly elevated in diabetic rats compared to control. Thrombin activity measured in the sciatic of the diabetic rats was significantly inhibited by in-vitro addition of NAPAP and TLCK compared to untreated diabetic rats. Data are represented as mean±SEM. *p ≤ 0.05, **p ≤ 0.01. Number of samples: control-saline (n = 3), control-NAPAP (n = 3), control-TLCK (n = 3), diabetic-saline (n = 3), diabetic-NAPAP (n = 3), diabetic-TLCK (n = 3).

Thrombin activity in the sciatic nerves was measured ex-vivo. Thrombin activity levels were standardized according to control group which included control rats treated with saline, NAPAP and TLCK. Thrombin levels in this group were defined as 1. Thrombin activity was found to be significantly elevated in sciatic nerves of diabetic rats compared to untreated diabetic rats (1.91±0.0.36 fold increase, p≤0.01 Fig 1). The increased thrombin level was significantly inhibited in the presence of NAPAP or TLCK (1.28±0.07, 1.16±0.13 fold increase, p = 0.028 and p = 0.009 respectively, compared to diabetic rats, Fig 1).

### Metabolic parameters in STZ diabetic rats

**Fig 2:**
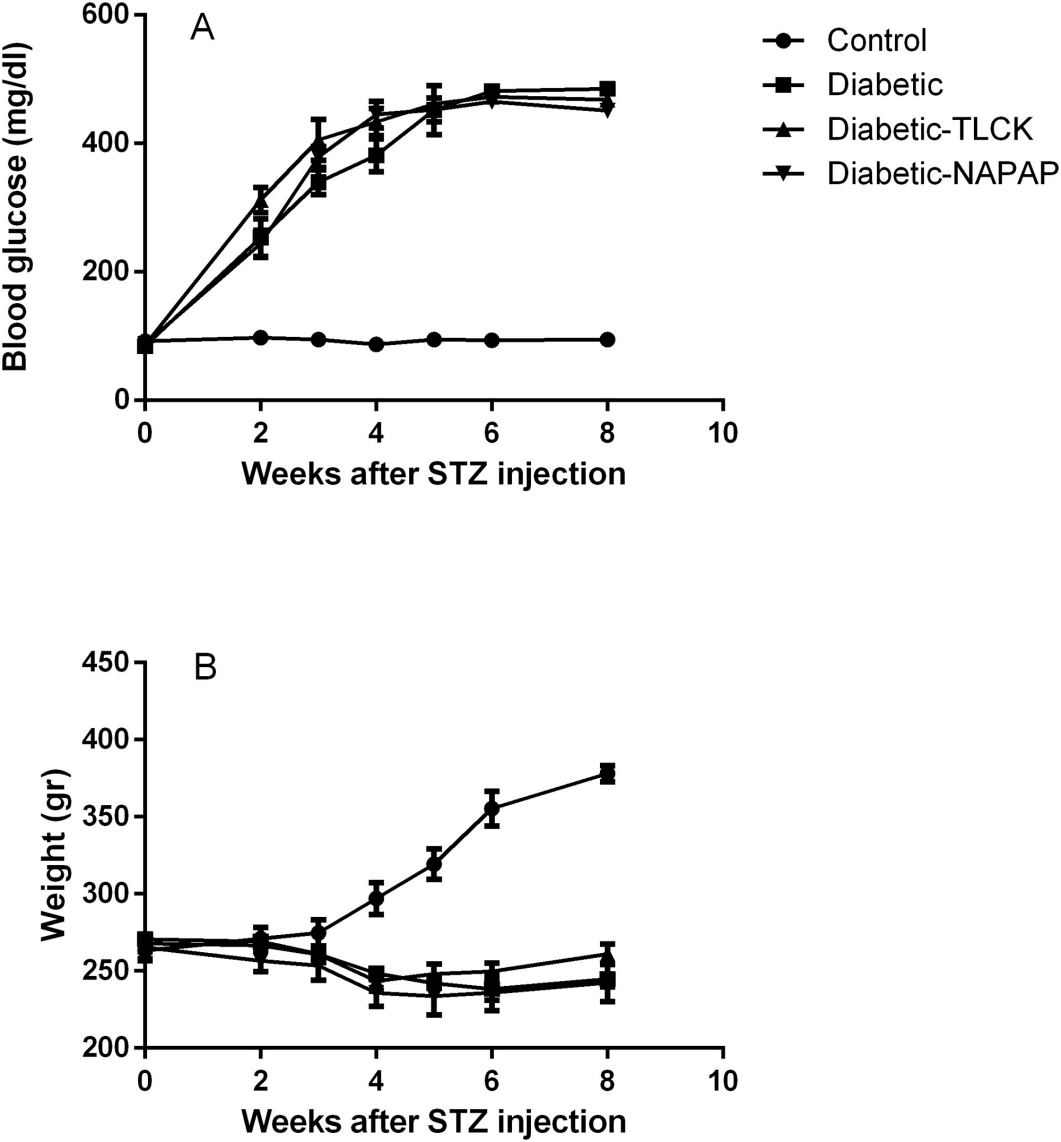
Metabolic parameters are not improved by the treatments. (A) Blood glucose levels and (B) Body weight in the control-saline, diabetic-saline, diabetic-TLCK and diabetic-NAPAP treated groups. Data are represented as mean of weight (gr) and blood glucose (mg/dl)±SEM. Test performed as described in Methods. Number of samples: Control-saline (n = 5/4), diabetic-saline (n = 5), diabetic-TLCK (n = 8), diabetic-NAPAP (n = 6).

STZ treated rats showed an approximately five-fold increase in blood glucose levels compared to control rats (481±11.82 and 94±2.41 mg/dl six weeks following STZ injection respectively, p<0.01, Fig 2A). Blood glucose levels were consistently elevated throughout the study period. Diabetic rats showed a significant decrease in body weight compared to controls (245±6.57 and 378±4.67 gr, respectively, p<0.01, Fig 2B), at six weeks following STZ injection. Treatment with either NAPAP or TLCK did not change blood glucose levels or body weight significantly (Fig 2). None of the animals died during the course of the study.

### Electrophysiological studies and heat response after treatment

**Fig 3:**
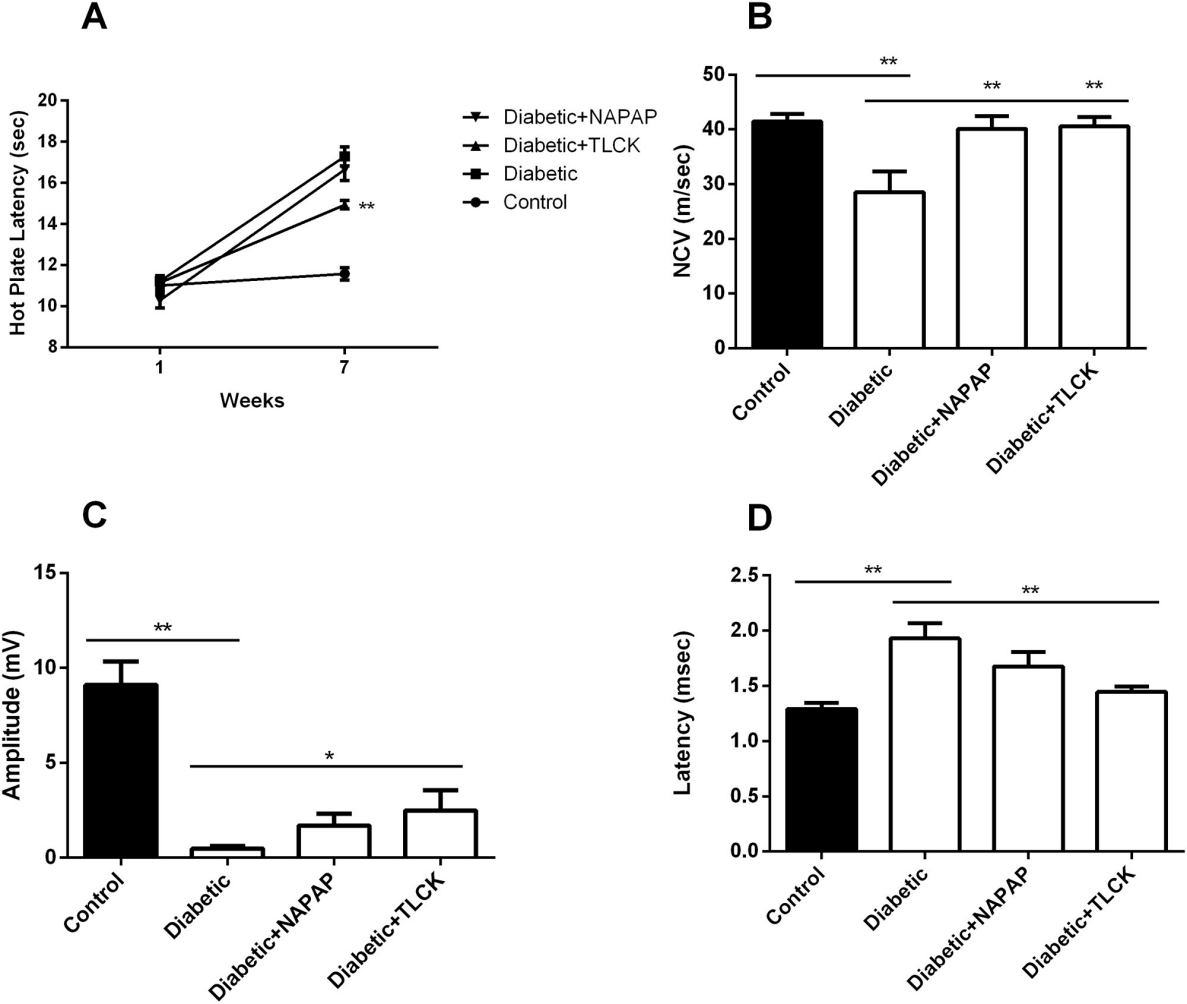
Heat response and nerve conduction impairments are improved by TLCK and partially by NAPAP. (A) Hot plate latency was significantly longer in diabetic rats compared to control (week 7). TLCK but not NAPAP treated rats had significantly shorter hot plate latencies compared to untreated diabetic rats. (B) Nerve conduction velocity was significantly decreased in untreated diabetic rats, and was preserved by treatment with NAPAP and TLCK. (C) CMAP amplitude was significantly reduced in diabetic rats as compared to control. Amplitudes were significantly increased in TLCK treated diabetic rats compared to untreated diabetic rats. NAPAP treatment showed a non-significant trend towards improvement in amplitude. (D) Latency time was prolonged in diabetic compared to control rats. Latency was preserved in TLCK treated rats. Data are represented as mean±SEM. *p ≤ 0.05, **p ≤ 0.01. Number of samples: Hot plate control (n = 8), diabetic-saline (n = 8), diabetic-NAPAP (n = 8), diabetic-TLCK (n = 7). Amplitude control (n = 4), diabetic saline (n = 5), diabetic-NAPAP (n = 5), diabetic-TLCK (n = 5). Latency control (n = 4), diabetic-saline (n = 5), diabetic-NAPAP (n = 5), diabetic-TLCK (n = 7). NCV control (n = 4), diabetic-saline (n = 5), diabetic-NAPAP (n = 5), diabetic-TLCK (n = 8).

Latency time on the hot plate was significantly prolonged in untreated diabetic rats compared to controls (17.29±1.16 versus 11.57±0.73 sec, p<0.0001, Fig 3A). Treatment with TLCK showed a significant reduction in hot plate latency time compared to untreated diabetic rats (14.93±0.49 sec, 17.29±1.16 sec respectively, p = 0.0003, Fig 3A) indicating improved temperature and pain sensation. NAPAP treatment did not achieved a significant reduction in this measure compared to untreated diabetic rats (p = 0.6, Fig 3A).

Conduction velocities measured in NAPAP and TLCK treated rats were significantly higher compared to the untreated diabetic rats (40.1±2.7, 41.95±3.9 m/sec and 28.5±2.8 m/sec respectively, p = 0.009, p = 0.01, respectively, Fig 3B). TLCK treatment significantly increased proximal amplitude (2.48±0.8 mV compared to 0.48±0.14 mV respectively, p = 0.03). Treatment with NAPAP caused a non-significant improvement in proximal amplitude (1.68±0.64 mV, p = 0.22, Fig 3C).

TLCK treated rats showed decreased and normalized latency as compared to untreated diabetic rats (1.45±0.05 msec and 1.93±0.14 msec respectively, p = 0.0019, Fig 3D). NAPAP treatment showed a trend to improved latency as compared to untreated diabetic rats (1.68±0.13 msec and 1.93±0.14 msec respectively, p = 0.111).

### Sciatic nerve PAR1 nodal localization in diabetic rats

**Fig 4:**
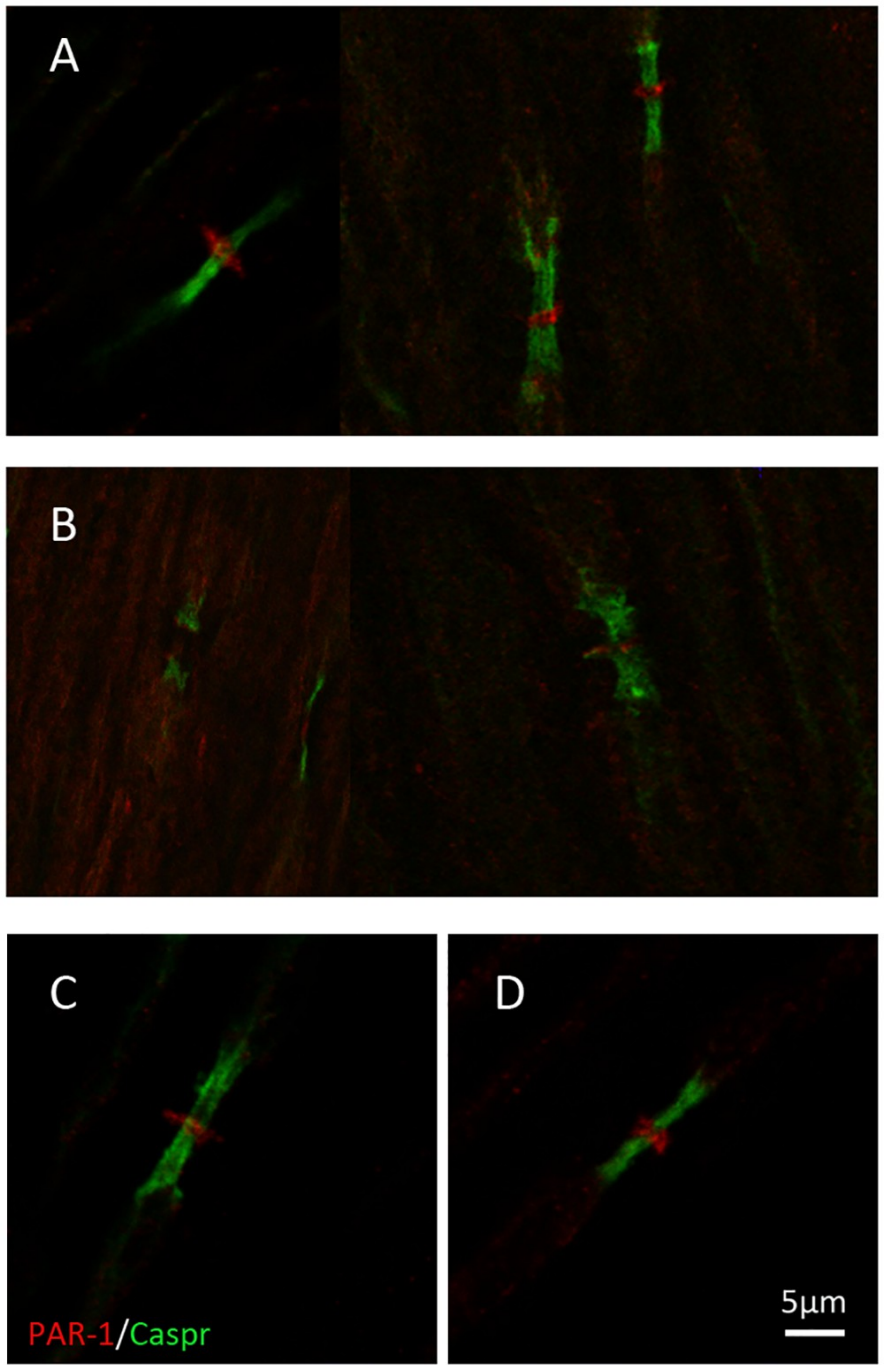
Nodal morphology is damaged in sciatic diabetic rats and preserved by NAPAP and TLCK treatments. Representative sciatic nodes of Ranvier from (A) Control, (B) Untreated diabetic, (C) Diabetic treated with NAPAP and (D) Diabetic treated with TLCK rats. The sciatic nerves have been double-stained for PAR1 (red) and Caspr (green). (A) Control rats’ sciatic node stained for PAR1 in a ring-shaped pattern between two paranodes structures stained by Caspr. This staining disappears in untreated diabetic rats (B), and is preserved with thrombin inhibition (C, D). Scale bar: 5μm.

PAR1 presence and nodal structure in rats’ sciatic nerve sections were examined by immunofluorescence (Fig 4A–4D). The paranodal axonal region was labeled with a specific antibody directed at Caspr. Antibody against PAR1 showed its typical ring-shaped staining at the node of Ranvier (red staining, Fig 4A). In diabetic rats, PAR1 staining in some of the nodes was completely absent while in others it was disrupted (Fig 4B). Distortion of PAR1 in diabetic nerves (up to 4-fold increase in length) was found in 26/30 versus 1/30 in controls (p<0.0001, Fisher’s exact test). PAR1 nodal structure was preserved in NAPAP and TLCK treated compared to untreated diabetic rats (19/30, p = 0.047 and 20/30, p = 0.028 nodes displayed abnormal nodal structure respectively, Fig 4C and 4D).

## Discussion

Our study demonstrates the importance of the thrombin pathway in a rat diabetic neuropathy model. Elevation of thrombin activity was measured in the diabetic sciatic nerve. Thrombin activity elevation was accompanied by destruction of the nodal histological structure and impairments of thermal latency and electrophysiological nerve conduction. Treating diabetic rats with both specific and non-specific thrombin inhibitors prevented development of electrophysiological, biochemical and histological abnormalities, and the use of the non-specific serine protease inhibitor TLCK improved thermal latencies. TLCK improved latency in nerve conduction studies as well. NAPAP was kept at a dose in which it provides specific thrombin inhibition, and did not significantly improve the results of thermal latencies. It is important to note that the STZ neuropathy model is in use for studying painful small fiber neuropathy as well, but in earlier phases of the disease [19]. The effect on thermal sensitivity represents the distal small fiber being affected early in the disease. Therefore, it is possible that the late timing of the treatments is one of the reasons why NAPAP did not significantly improve thermal latencies.

Improvement in latencies and histological measures seen with TLCK treatment may be due to TLCK extensive effect on other serine proteases rather than thrombin. Another possible explanation for TLCK positive effect is its neuroprotective properties. TLCK has been previously shown to be neuroprotective [20], via a mechanism involving reduced glutamate toxicity in the mitochondria [21]. As described in Methods, TLCK was used in high doses, and it is difficult to differentiate which effect has contributed directly to thrombin inhibition. Although the mechanism behind TLCK effect on nerve conduction and morphology may be more complex and include both anti-inflammatory effects and a direct effect on nerve conduction, the effect of NAPAP is more specific. Therefore, its mechanism of action may be related to the direct improvement of node of Ranvier function as can be seen in its morphology (Fig 4) and in nerve conduction velocity (Fig 3), suggesting that the destruction of nodal architecture may be related to the measured conduction abnormalities. Comparing TLCK and NAPAP, the relative efficacy on fiber number is also reflected in the effects of these substances on the amplitude of muscle responses, which was similar.

Our study has some limitations. Due to electrophysiological considerations, the electrophysiology was done on the tail nerve, while thrombin levels were measured in the sciatic nerve. We believe that both nerves represent peripheral neuropathy. Further evaluation of the PAR1 pathway expression is needed. This was out of the scope of our study due to limited number of animals and the need to subject some of them to histopathology. Further evaluation using quantitative measures (such as western blot) is needed in the future. In the present study, we focused on searching a specific pathology in the node of Ranvier, and therefore preferred the qualitative over the quantitative methods. In our study we used relatively young rats at the age of eight weeks, when the animals are not fully mature. Although young, this is the accepted age for this animal model, and it is a good representation of the young onset of type 1 DM [22].

Specific measurement of thrombin activity from nervous system tissues is a complex issue. The substrate we used for detection is peptide-based and prone to cleavage by other non-specific proteases and peptidases, producing a background noise activity. Using a combination of inhibitors of non-specific proteases as previously described [15] a clear and measurable level of specific thrombin activity was measured and inhibited by the specific thrombin inhibitor NAPAP. Using this criteria, there is a validated increase of thrombin activity in the diabetic sciatic nerve compared to normal nerve.

Elevation of thrombin is in-line with previously observed nervous system disease models such as stroke [23], mild-traumatic brain injury (mTBI) [24], nerve crush [25] and the finding that thrombin activity influences synaptic function [24,26–28].

Coagulation and inflammation are known to modulate each other. Thrombin provides a link between inflammatory and hypercoagulability conditions [29,30]. The increase of thrombin activity in diabetic rat sciatic nerves is consistent with previous measurements in the blood of diabetic patients. Thrombin activity in the blood and glycemic control are known to be correlated [8]. Thrombin has been previously described as a contributor to the pathogenesis of diabetic neuropathy by hypercoagulability [9,31] and is involved in damage to other systems in diabetic patients, such as the kidneys [32] and the cardiovascular system [33]. Diabetes, therefore, encompasses both hypercoagulability and hyper-inflammatory properties.

We employed treatment with exogenous thrombin inhibitors to further establish the importance of thrombin in the pathogenesis of diabetic neuropathy. This showed beneficial effects. The treatment preserved electrophysiological nerve function and PAR1 morphology at the nodes of Ranvier. These results indicate that excessive thrombin activation at the node of Ranvier induces nerve conduction dysfunction. Many studies conduct electrophysiological tests on the sciatic nerve. However, based on our previous work we decided to conduct the electrophysiology tests using the tail [17]. This approach holds some clear advantages over using the sciatic among them is the length measurement which is more accurate and reproducible.

Since diabetes involves a hypercoagulability state [34], thrombin inhibition may also contribute nerve well-being by blood flow improvement. Although improvement of blood flow may help nerve function, no data indicating that anti-coagulation therapy improves neuropathy in diabetes patients has been published. This observation suggests that nerve function improvement with thrombin inhibition is mediated by factors other than blood supply alone. The connection between thrombin pathway hyperactivity and development of diabetic neuropathy is still unclear, and further research is needed in order to shed light on the subject.

Current treatments of diabetic neuropathy are not aimed at reversing the pathological process underlying the disease nor the metabolic related parameters. The targeting of the thrombin pathway in diabetic neuropathy is novel and feasible since these patients have essentially a hypercoagulable state and are thus amenable to thrombin inhibiting medications. Thrombin inhibition carries the risk of bleeding, and indeed, thrombin inhibitors used in this study caused a bleeding tendency in the laboratory animals and was detected only post mortem (not shown) with no clinical evidence during the experiment. TLCK caused more bleeding than NAPAP, most likely due to its wide range of activities and the high dosages used during the experiments. Novel oral anticoagulants are already in clinical use. NAPAP has similarities with Argatroban, which is available for use. This calls for further research regarding expansion of the current indications for it and for other thrombin direct inhibitors use as a possible future treatment for diabetic neuropathy.

### Conclusions

Our study demonstrates the important role of the thrombin pathway in diabetic neuropathy. Thrombin activity elevation, was measured in the diabetic sciatic nerve, and was accompanied by destruction of the nodal histological structure and impairments of electrophysiological nerve conduction. Treating diabetic rats with a specific thrombin inhibitor improved their nerve conduction measures and prevented biochemical and histological changes, suggesting thrombin inhibition as a possible treatment strategy for prevention of diabetic neuropathy.

## Supporting information

S1 Dataset All experimental raw data. This file includes the values behind the means and the SEM, and the data used for the graphs.
(PZFX)
